# Specialties and Their Advantages

**Published:** 1882-11

**Authors:** Geo. A. Mills

**Affiliations:** Brooklyn, N. Y.


THE
### AMERICAN JOURNAL
OF
### DENTAL SCIENCE.

Vol. XVI. THIRD SERIES—NOVEMBER, 1882. No. 7

### ARTICLE I.

### *Specialties and Their Advantages.*

BY DR. GEO. A. MILLS, BROOKLYN, N. Y.

[Read before the American Dental Convention, at Saratoga, Aug. 9th, 1882.]

Mr. President and Gentlemen:—It is not my purpose to argue the question regarding the propriety of specializing various departments of labor, but to enter at once the arena of history and to state the accumulated evidences found in all the departments of divisible work. Nature has set the example throughout the cosmos of creation. Look where you will and the query will arise to the thoughtful mind—why is this so arranged? And when the answer is found it invariably acquaints us with the fact that it is arranged to be so for special purposes. Nothing is created in vain. All the multitudinous divisions of the animal kingdom into species are so made to promote special ends. Man, who has been placed upon the highest plane, has been endowed with intellectual capacities to perform special work, showing it is a special purpose for wise ends. When this is made clear we shall be satisfied; until then let us wait. The inquiring mind seems to know no satisfaction; the possibilities being exhausted in any given direction, new zest is applied to researches in fresh and unexplored regions. To our finite comprehension there seems to be no limination to the work which the human mind can find to do. All nations have done work, conscientiously or otherwise, that specialized itself in the history of accumulated civilization. Nations have done, and still continue to do, their governmental work by a system of prescribed laws. All this is working out special ends, and the ultimation will be productive of good results to mankind. Mental activity has had its special representatives in all the known history of the race; the recorded evidence of this fact exists, impressed on the works of nature by unknown hands, and in some instances in unknown tongues, yet to be deciphered by the specialists in the department of languages. We can say, as a general remark, that the numerous departments allied to the interests of mankind are special, and each has, and will continue to have, its special end in view, with a trained hand to conduct the general work. Under this head there will be subdivisions of labor by specialists. In our day we have three divisions of special labor, called professions, that have their historic value, which reveal potencies that exert an influence resulting in beneficient products to the mind and body. These divisions are divinity, medicine, and law. With the proper knowledge and its legitimate application, there lies within this tripod the solution of the problem of the highest development of civilization. The first will lead to mental foresight, the second correct the disordered physical condition, and the third establish us on an abiding foundation of harmonious living. These calling are associated with many departments of collateral research, and there scarcely seems to be an end to the devices of men to solve the hardest of all questions: What is truth, and where shall I find it? Through all ages the needs of mankind have, in due time, presented their demands in accord with development, and out of all these emergencies a deliverer has appeared to appease the desire. In the language of our old and familar proverb, we find the fact that has always been and always will be true—"Necessity is the mother of invention." The noblest specimens in representative labor have had their origin in humble spheres, from the Man of Nazareth to our own martyred Garfield.

Genius needs the stimulus of necessity. The public owes much to that necessity which has so often spurred genius into action. While we see much to admire in the noble and self-sacrificing life of a genius, yet his greatest uobility will only be revealed by the pure and white light of those rarefied atmospheres of his future life within the realm of unselfishness.

Genius is not uncommonly found in company with specialized labor; but more often special work is found allied to motives that are not compatible with the life of a genius. Perhaps it may be better that a low motive prevail than that there be none at all. Yet I am sure that in this unparalleled age of progress, in which we are being sped on the wings of electricity, whirled at one moment into seeming impossibilities, and the next, all doubts being dispelled, we can afford to be patient, that out of all these efforts there shall come success.

What wait we for but the crowning of mankind with peace and good-will toward men ? Yet while we wait it is not without hope. America is to be the arena in which this grand problem is to be worked out. For to-day indicates that, were we wanting for material with which to grapple with the problems of life, we shall have it, for the representatives of all nations are flocking—"as doves to their windows"—in almost countless numbers to our shores. They are bringing something of almost everything, and with liberty, without license, these commodities are to be passed through the process of fermentation, out of which will come the spirit of reconciliation which has been brewed out of untrammeled intellectual thought and action. Then to no other land can we look as offering so much encouragement to progressive specialized labor. While there is much that should admonish us that whatsoever we sow that we shall surely reap, yet I think much will be accomplished that shall soon soften the harsh grasp of the avaricious, while at the same time increasing intelligence shall stimulate others to greater efforts for a better position for themselves by the practice of a greater frugality. Honest, cheerful labor will, I predict, do much to lessen the desire, so destructive, for artificial living. Wealth is not at all wrong, neither is labor all wrong.

With this prolonged introductory, if it may be so termed, I will now note some of the most prominent results of systematic labor by specialists. In the financial department we have some very noted specimens, such as W. H. Vanderbilt and Jay Gould, who are perhaps the most prominent. They stand before this young country almost as miracles, repeating that dazzling, wonderful story of our youth, the Arabian Nights. I am not one that counts them the worst of sinners. They are doing for this country in a decade a work that it would take centuries for associations of men to do. By the word of mouth they dictate into existence, almost in the twinkling of an eye, a projected artery across a vast expanse of territory heretofore abandoned to the wild beast and the cactus; and ere we are aware of the fact, they are utilizing this ponderous channel by sending to these new territories thousands of pilgrims who come seeking homes in this great land of liberty, of which we sing, there to thrive and become allied, in the coming years, to all that makes us a prosperous and mighty people. These financial giants specialize their great and fabulous wealth by gigantic enterprises. If it were not for such avenues how would these myriads of human beings be distributed upon the countless thousands of unused acres in our land? It would seem, as we look in astonishment upon these determined, bronzed countenances that we see almost daily flowing out of the great railway caravansaries that jut themselves into the very waters which ebb and flow between us and these unlimited countries of Europe, as it were, scooping them up on our hospitable shore, it would seem, I say, that these men who are projecting so mightily were, with their energy and millions of resources, raised up to do this mighty work of advancing civilization. Cannot we wisely halt in our general habit of criticism, and spare a little, to see what the coming days shall send? Human judgment is so very easily made to prejudge in all matters of public doings that its haste often prevents the best deductions. A question that may well be asked, and which, as you will see, is not foreign to the subject we are considering, is: Can the march of civilization to its destined end traverse the rough and untrodden way of barbarism without disturbing some of the lower conditions of existence?

I might continue on through the varied departments of human labor, and divide and sub-divide from the highest to the lowest, but I will hasten to make the application in the interests of the department to which we are devoted. And, in passing, we will not omit to notice a few who have made themselves prominent in these varied departments, more particularly in our time.

In mercantile life we record the names of A. T. Stewart and H. B. Claflin, both of which find no rivals, and who are classified as merchant princes. Concentrated energy has specialized their conduct, and their methods of trade, wisely arranged, have resulted in munificent rewards.

The profession of literature has, in our time, given us not a few names of distinguished celebrity, among which are the poets Longfellow and Bryant, who have not only sung out to us the language of their own souls, but that of thousands of others. And they rest from their labors, well done.

We call out from the score or more of authors, the names of Irving and Holland, both not soon to be forgotten. Such a philosopher as Emerson has left the general impress of his daily notes, which were gently wafted into his fertile mind by day and by night, and the coming time will record their value, and pay tribute to his memory in the utility of his richly garnered pearls. Also the names of Webster and Evarts will long illuminate the roadway of the law; and the day will be very far from this when the power of that mighty intellect enshrined in the form of Horace Greeley, and inspired by an honest heart, shall drop out of memory or cease to inspire the press to high attainments. His example shall be even more lasting than any monument that men can erect.

To speak of the long list of names that have marshaled the dogmas of theology, and select a trio as shining examples, would doubtless put me in the attitude of a sectarian, for the divisions are so numerous, and favoritism runs to such extremes, that it is not an easy task. And yet it is true that such men as Beecher, Chapin and Bellows, have, in our time, wielded a mighty power for the good of their fellows, that will be lasting in human experience, animating thousands in their daily work, and aiding and advancing Christian civilization. They have cast fruitful seeds into much broken and mellow soil, which must spring up and bear fruit to the honor of the race.

Among the centuries a host of names have been emblazoned on the pinnacles of fame from the division of labor classed as healing art. If we garner from out of those that are truly representative of our new and growing country, I find myself, as in the last situation, with a difficult task in hand, for I am reminded of the *pathies* and *no-pathies*, the specialist and the generalist. To speak of the generalist would seem to be apart from the direct purpose of my paper, but that I may harmonize, I shall hold that he can only excel by specializing for general purposes; and I shall not expose myself to much, if any, criticism, when I say that few are endowed, in any large sense, with capabilities that will or can mature a generalist. Such as a Mott, and a few of his stamp, are safe to memorialize, but, in the division of the many departments, we find active in this day the name of Joel Parker, and Pancoast and

Warren, who are distinguished quite above the average in the specialty of surgery, which is, as you are well aware, a youthful branch, as compared with the age of general medicine. Sims and Thomas have made the field of the gynecologist one of mercy and relief to that class of sufferers so common, and we, who have no knowledge (by experience) of them, can well give thanks for their contribution in the alleviation of such peculiar trials. Agnew and Knapp doubtless have gained a reputation as oculists and aurists, which is freely accorded to them as ranking as high as any, if not the highest, among their fellows.

The time will not allow, without weariness, to fill out to completeness the names of the numerous representatives of other specialized departments, and only to speak, in passing, of the neurologists, associated with the names of Brown-Sequard, Seguin, Beard and Hammond; the orthopedists, Taylor, Sayres and others, and lastly, I will refer to him who, as yet, may not be crowned by the general fellowship of his calling, but who is destined to be accorded a position as a specialist in the department of histological research of the animal tissues, second to none in this country, or, I predict, in any country, Carl Heitzmann. Yet but thirty-six years of age, a native of Hungary, schooled in medicine in his native land, becoming associated in Berlin, Germany, as a draughtsman with parties employed in the department of governmental work in the scientific field of research, he was, by a coincidence which we would often denominate providential, by the death of the head of this department, called to supply the vacant place at a salary amounting, in our money, to about ten thousand dollars. Coming to be cognizant of the work of the several specialists employed in this field, he discovered that in the acceptance of the protoplasmic theory all had settled down upon the decision that this body of material was a structureless one. The thought struck him: is that a consistent view to accept? It was an inspiration to him to try and prove the untruth of such a meaningless and vague idea. As he said, "When I took up the study of protoplasm in 1872, I had before me a blank book." But to-day he has his reward in the acknowledgment of the ablest microscopists of the world. Seven years circumstances favored his coming to this country, with hope of greater liberty in the pursuit of his work. Although commencing his labors in a quiet and unpretentious manner, his field of labor has steadily been on the increase, so that now his laboratory in New York City is a place of great resort by those engaged in histological research.

To this famous student and a few co-laborers from out of our ranks, (for which great credit is due to Dr. W. H. Atkinson, who introduced him to the notice of our calling) we owe much; and, gentlemen, the pages of our literature are now bearing testimony to the fruits of these labors, and we are able to say that in no branch of scientific research associated with the healing art are the histological and morphological portions of the human anatomy better understood than that of the human teeth and their connected tissues. The confirmed knowledge of the minute anatomy of these tissues has come into our possession in less than a half decade, by the energies of Carl Heitzmann and his eminent co-laborer, Dr. C. W. Boedecker, of New York City, a member of our calling whom we delight to honor. This feature of my remarks brings me to the part of my paper that will be of special interest to us. We are well aware of the attitude of this our department of dentistry at the time of its conception. Generated in lonely surroundings and nurtured on a low diet until it was evoluted into a more ambitious stage of existence, and going on until it reached the times of such a benevolent spirit as Chapin A. Harris, who caught the inspiration to provide a more befitting atmosphere for its future life. And we may well do honor to such a man, that with so little he did so well. By special energy and concentrated labor with wise associates, our department for the allevi- of human suffering was first cradled in the city of Baltimore, by the institution of the Baltimore Dental College, under a charter given in Maryland in 1839. And the alumni of this college embraces in its list not a small number who have occupied positions of prominence both in this country and in foreign countries. From this special endeavor a nucleus of energy was created, which has been disseminating its influence gradually and certainly, so that to-day we number in all fourteen institutions. All this is the result of honest, earnest and special effort. The progress made in our calling has not been surpassed in any other during the last twenty-five years. Doubtless the feature of associated effort has given a direct impulse by developing the latent forces acted upon so advantageously by the fraternal atmosphere of association. This body has had a signal influence upon this work. It commenced the labor of bringing (in large bodies) out of chaos into concert of action, from which advancing thought has grown to a more defined method of labor. Although it may be thought by some that it has outlived its mission, yet it does not follow that if there be a feeling of devotion to the institutions of a more democratic method of governmental action, it need be discontinued ; it is certainly not hurtful, but cannot be anything but helpful, for the fraternal influences alone will develop a bond of union that is not without its expression for usefulness. We have not outlived yet our mission towards the unorganized mass of practitioners. I declare myself here and everywhere an integral part of the department of the whole healing art, and on the side of helpfulness to any and all that I can assist. The day has not yet come to draw the lines too strictly, and it would be more becoming, I think, for some who figure so diligently as law-makers to be a little modest It is better, and I think it is profitable, that occasionly we look back to the pit from which we were digged. I am not by any means opposed to any and all work of our several societies who have in mind sincerely the elevation of our profession. But I do think that the transactions of some of these societies are fostering more of a spirit of arbitrary seclusion, fostered under the guise of pretension, that, if continued, will give rise to the suspicion that the best interests of the profession will not be attained; for instance, the creation of a degree by the New York State Society, and this degree put side to side with those given by institutions chartered by the various States, and one particularly in this State. It is a lack of good judgment, certainly, and if it is continued will be a shame and disgrace. No high-minded man with a little reflection can but see the shallowness of such a degree as this M. D. S. issued by a State organization. What would common sense say of such a procedure applied to law and divinity, or to medicine generally? It would stamp it a sham. This may be thought a little general in its application, yet I answer no; it is a work of special labor to see that it does not long deface the creditable advancement of our calling. It behooves every thoughtful man to give emphasized expression until its work of misguiding shall come to an end. Does any one think I am a lonely voice to speak against this thing? Nay, nay, I know whereof I affirm. More, I know the thought, in no small degree, of the representative portion of the profession regarding this, but it will take a little courage to throttle this mistake. It has unfortunately, and, I believe, unthinkingly, been attached to the signatures of not a few worthy men, and this fact makes it all the more serious in its results, and this very class of men should see to it that they do not countenance a work that stands so utterly in opposition to all that is progressive. For bodies of men to form themselves into exclusive alliances, shutting out such as do not chance to hold any degree, seemingly giving out the idea that attainments only can be found behind a degree, is very weak indeed. I honor all degrees creditably secured, but I say that in these days of such rapid advancement in the acquisition of real scientific knowledge, these distinctions based on such puerile pretensions are certainly not becoming any body of men who are truthfully desiring the elevation of their calling. I assume the liberty of this digression, knowing that I am before a democratic body; yet I shall claim that what I have said has an immediate bearing upon what I propose to say further in this paper.

You have doubtless noticed, as I have, the fact that few men are endowed with capabilities sufficiently fertile to enable them to excel in any large sense as generalists. We are not unfrequently reminded by this fact of the reasonableness of a greater division of labor, and to some extent it has been effectually put into preatical use. And this evidences the importance of urging the value of more numerous subdivisions, with the hope that more fruitful results will follow; and I think all of us should seek to do all we can to mould the future character of our calling in this direction, and aim by word and deed, in concerted action and in our general intercourse, to so mature the purposes of such action that it shall be instilled into the minds of those who shall choose our vocatiou. These should be impressed to follow the branch of labor for which they are best fitted. For instance, one has a particular taste and fitness for fitting teeth, another is possessed of patient ingenuity, adapting him to occupy the field of correction of oral deformities, for which mechanical appliances are requisite. This may or may not embrace the portion of service allied to the construction of artificial teeth, yet my theory is that the field is so fertile, and so much in need of a higher grade of culture, that it could wisely and profitably be made to become more beneficial in its results. And I would say the same in connection with the more prominent features of surgical services. All these divisions require, yea, they demand (that they may result in the greater good) the most liberal assistance of all the scientific allies that will aid in the largest acquirement of skill. And here I propose to make particular mention of some who have, in no small degree, acquired merit, and have received a reward that compensated somewhat for their efforts, accompanied with many difficulties which will not as much distress those who may ally themselves to these special departments hereafter.

How little we realize the cost of foundation work, and how little we understand, in any large sense, or appreciate the motives of those inspired to do this preparatory work.

No man has been more intimately identified with the special life labor of infusing a spirit of progression into our calling than Dr. W. H. Atkinson, of New York. He has been a Prophet, Priest, and King. As you all know, with an endowment which has shown remarkable evidence of unlimited resources, he has been an Elijah to us, fearing no obstacle of opposition. Some of you will remember his first advent before this body at a meeting in New Haven. He then came among us like an apparition, unshorn and unshaven, and was then termed the crazy Atkinson, the wild man of the West, etc. Now if you can cast a view from that day until now, you can behold what he has wrought. Any that have been at all cognizant of professional workings until this time can readily see his imprint on all that has marked the way of progress. By this I do not mean to infer that nothing has been done but by him, but that he has been largely the inspiration of most of that which has infused a spirit of enthusiastic zeal, is certainly true. Wherever the interest of this profession exhibits itself there his name is known and honored. Truly his reputation is not alone national, but world-wide. May his last days with us be his best.

We cannot fail to notice so valuable a contributor to the advancing status of our service as Dr. Garretson, of Philadelphia, who has labored so assiduously to raise the standard of special surgical skill to a position that is second to no other department of the healing art. It is doubtless true that he has no pear as an oral surgeon, and we can only be proud of such an accession. The establishment of the Hospital of Oral Surgery, in connection with the Philadelphia Dental College, is certainly one of the most signal steps in advance that has been made in connection with practical instruction, and offers advantages that should tempt cultured men to this special field of service, for I know of none that holds out more flattering opportunities for large success. It is truly gratifying that the indication of the demand for advance in this work is being vividly impressed on the profession.

Another feature of practice has fallen signally low, and done much to dim the earlier lustre that had been doubtless truly accorded to the branch that has been termed mechanical dentistry. But to-day it is rallying for a better expression, I predict, under a new and doubtless more befitting term, prosthetic dentistry. I do not mean to detail the decline of this department; I had much rather hasten to signal the prospect of a higher grade of practice. The desponding position of this department has commenced to yield its results, and is already indicating that we are nearing a grade of practice associated with higher attainments. Yet these hopeful signs are not the results of sudden chance. Thanks to the nobility of untiring genius that has not bowed the knee to Baal, but through years of patient toil, encountered by many sad and heartsore disappointments, cast down but not defeated, stern necessity has stimulated to higher purposes, and year by year diligently pursuing, and learning to labor and to wait until the reward of honest purposes should appear.

Gentlemen, to whom do we owe a debt of gratitude in this direction more than to any other man? To our worthy and world-honored brother and co-laborer who sits with us to-day, Dr. John Allen. My dear sir, you have fought this warfare; you have never faltered even in the hour of seeming defeat; when many would have been discouraged you rallied your little all of efforts—and they were little of this world's goods—with a faith that looked over and beyond, to a future that should reveal a more hopeful time. My dear brother, in your fast declining years, though indicating that physical infirmities are fastening themselves upon you, and you are reminded that what you do must be done quickly, you will soon rest from your labors. But most assuredly your works will remain; and those of us who may survive you will sincerely accord to your memory honor and praise for the noble and self-sacrificing work you have accomplished. And it is a comfort to know that, although we commit our works to other hands, that which is of value will remain. We are so constituted that we do derive satisfaction in the rewards coming to us in this world for meritorious services, and you have not been overlooked in this respect. You have, certainly, in those richly ornamented gifts presented to you by all the national and international exhibitions, the world's evidence that the attainment of skill has been of a grade much above the ordinary; and we can but hope that they will find some secure place of deposit that will be, in the near future, provided by the munificence of some one or more of our calling; for certainly they can but be estimated, in no small sense, as an incentive to those who will follow us.

I have noticed that there has recently been published and distributed, by authority of our Congress, the official reports of the United States Commissioners of the Paris Exhibition in 1878, and among other things mentioned it says. under the head of American Dentistry: "The exhibits in this department were adjudged far above all others, and the award of the grand prize medal was accorded to Dr. John Allen & Son, of New York City." Certainly we can truthfully congratulate you and rejoice with you; and we also are able to deduce from all this, tangible testimony of the advantages resulting from special service. There is already enough evidenced in what I have said to inspire the purposes of all, and also to create an incentive that will impress upon us the importance of greater exertion in the department of special *surgical* labor.

I have alluded to the signal evidence of a decided upward advance in the department of prosthetic dentistry, and it may be well to notice in what direction it is. In the first place, by a greater desire shown to conserve the natural features of the face by a decrease in the practice of so ruthlessly removing useful teeth by extraction; second, in the marked increase of retaining the roots of teeth from which the crowns have failed; third, a return in a larger percentage to crown setting upon these roots, associated with improved facilities which give greater assurance for their more uniform comfort and durability. We can but notice with what enthusiasm special effort is working in this direction, and helping in no small sense to answer that question, which has an important bearing upon the work we have in hand: How shall we meet the masses at a price they are able to pay? For, view this as we may, the poor and the penurious we have with us always. Probably the two most prominent improvements, and the two that will become the most universal because of their greater simplicity and inexpensiveness, are the Richmond and the Bonwill crowns. Their introduction has proved their supposed value, in a very large sense, by their popularity in so short a time. The Bonwill crown will doubtless find a more ready application in practice at the outset. Both of these methods have objections raised against them. The Bonwill is the least expensive, considering its first cost, yet I think they are far more liable to require frequent repairs. I think in the Richmond method, and in connection with it, there has been devised an improvement that should attract our attention.

I have not yet, only in a general way, alluded to artificial teeth inserted on plates coming in contact with the mouth, Though there will be cases, from the necessity of which we can as yet see no escape, yet we know, all of us, the disagreeable conditions produced by the contact of any and all kinds of materials with the mucous membrane of the mouth; vastly more so with all non-conducting substances, such as rubber and celluloid. These plates not only pollute the health of the mouth in no small degree, but they entail other difficulties which are unlimited. I refer to the changes that take place in the tissues, both softand hard. These changes are produced by absorption, and bring about untold complications and discomforts, associated with frightful contortions of the features of the face, carrying distortion of expression to such an extreme that it is a common remark among all close observers. How many, there are that exhibit a prematurely old face? Is there anything that the mass of human beings will hail so joyously as an escape from these direful consequences?

Now, there may be evils associated with all these methods, but the thing we most desire to do is to make choice of that which entails the least of them. Now, from what I have seen in quite a large number of practical cases, I am able to say that, in my honest judgment, there has never come to us such a boon of relief to offer to the world at large, as the method of attaching artificial teeth combined with the Richmond Crown. I do not consider that the objections urged against the plan of placing the gold band attached to the crowns under the gum at the neck of the root, are weighty enough to militate against the priceless advantages gained over other methods. This kind of work, you will understand, I am advocating in such cases as it is applicable, where, for instance, the intervening teeth are absent and intervening roots are present, so that by the application of the crown on the several roots, the spaces requiring teeth where there are no roots can be combined by a process of bridging. I am well aware that this endorsement (which is of my own free will) will be thought a somewhat singular procedure by some (considering my standing in the profession) because of the plan adopted by Messrs. Sheffield & Richmond to make this invention known in such a public manner as they have done, by a large expenditure of money through the columns of the leading newspapers of the country. But I am reminded again that I am in the presence of a body that has no prescribed laws indicating the methods of conducting a practice. Gentlemen, I am openingly and avowedly opposed to any and all codes of ethics, and have always advocated against them, so this cannot be ascribed tc me as any new impulse. I see more clearly than ever the absurdity of such prescription ; and I assert, *knowingly*, that the hearts of the profession are in spirit absolutely against the continuance of these laws. And I predict that the time is near when the current will be changed, and in my estimation it will do more to adjust the discriminations than anything else we can do. Allow me to ask, what moral right have any body of men to legislate against any man making known to the whole human race, if he desires to do so, that he has (or believes he has) a special service at his command. It is a singular fact that the Healing Art is almost, if not quite, the only calling that holds to such a relic, if it be one of heathenism. You will notice ? that the new organization that has come into existence, and which calls itself, inspirit ,the National and International body, have purposely, and in view of the progress of the age, kept it out of their constitution, and they have acted wisely, for men whose chief desire is scientific attainment have no taste or desire to quarrel with their co-workers on methods, but their first and only thought is knowledge. The quicker all proscriptions are removed, the sooner the public will arrive at a wiser discrimination in their choice. I state knowingly that the honest convictions of our best men are in sympathy with my expressed thought. Many are on record in accord with these views, and many more entertain them, as yet quietly, and I predict that the time is not far off when we will see them put into practice. Special labor in this field will bring its advantages in this direction.

The subject of pericementitis, with the Riggs' feature included, which means, in a practical sense, a specific disease of the membrane of the socket, and manifested in a variety of ways, more or less familar to many, viz., accumulations of lime with their admixtures; so-called tartar; inflammation of the gums, causing them to bleed by the slightest pressure,. being very tender without apparent cause, often very loose; marked destruction of the gums and falling away, frequently an exterior exudation of pus, associated with a sickly, fetid oder in the breath, more noticed by others that by the person in trouble; and, in not a few cases, a general disarrangement of the bodily functions, expressed by great lack of energy, low spirits, loss of appetite ; and with this exhausting condition of health the patients are wholly unconscious that it is attributable to this disorder in the mouth, while they are constantly appealing to their physician for relief, and to no effect. I am impressed that these facts must be brought to the minds of medical men. To commence such a labor I prepared and read, by invitation, a paper, in May last, before the Kings County Medical Society, in Brooklyn ; subject, " Pericementitis, its Manifestations in the Oral Cavity, and its Serious Effects upon the General Health." This has been published in their monthy journal, and I have had a reprint of a thousand copies for general distribution, which will be sent by mail to any who desire them.

The physician has it in his power to contribute much assistance in the line of relief that calls so loudly for help. In my judgment more encouragement will come from medical gentlemen, for the reason that they will more readily detect the peculiarities of this insiduous disorders because they are cultured in surgical perceptions. Experience assures me that specialized energy will bring the results sought, sooner or later, in this direction.

The institutions for teaching, in our calling, are becoming conscious of the demands pressing upon them for additional efforts, and they *must* be prepared to meet them, or they will see opportunities springing up about them that will entice those seeking our calling through educated channels. One thing is sure, they must keep an energetic thought in action, ready to cultivate the field that signalizes the certain tendency to special teachings for special purposes. So far, all of the teachers have fallen short of the duty they owe to the student and the people, by their neglect to teach the knowledge attainable in connection with the special disease I am emphasizing the importance of. The time will come when the schools of dental teaching will be compelled to give more than a passing notice to a subject which is of such vital importance to the people. What do the students that are passing out of our several colleges by scores from year to year, know of this cyclone of destruction that is so insiduously entailing untold distress upon the mass of human beings? Is there any evidence that is worthy of record that is manifested in the infirmaries of these institutions? I answer most emphatically, no; and there will be none until the subject is brought before the classes by some one who has some knowledge to speak with, and something that shall *inspire* the young men who are looking for just such opportunities as such a field of service offers, second to none other in the whole range of professional science. Such apathy as I see manifested by men of no small attainments in other departments, is quite incomprehensible, with the view I take of the duties of one, who assumes the responsibilities of a teacher. So far as I have been able, I have had one steadily growing purpose during my whole professional life, to make such use of opportunities that I might be fitted to dispense helpful service in a degree above the ordinary. How far I have succeed I leave for future decision. Fortunately, I came into the calling impressed from the first with a love for it, and now, in my twenty-eighth year of practice, my affection does not wax cold, nor have I ever seen a moment when I could wish I had chosen any other avocation. In this I have escaped the direful lamentations that have so often come to me in my associations: "I wish I had never heard of dentistry," "a dentist has no honor," etc. etc. Such members of the profession have been only barnacles upon it, and have done much to retard and hinder the advancement of those who desire to make it an honorable calling. Those men are found clamoring for the elevation of the profession by loud and frequent lip-service and a very meagre attainment of ability to serve the people with a service based on real intelligence.

I am well aware that my enthusiasm has made me aggressive in advocating my views, and I have learned that all progress is of its nature, so. Fortunately for my growth, intellectually and practically (at least) I have allied myself in my most intimate associations, with those who were my superiors, and, as I look over the last twenty-one years, I must say that if I could not see tangible evidences of a rich development I would then be compelled to admit that I have been a dull student. And all this enables me to bring to bear upon this specialty I have espoused, a degree of zeal and understanding that the nature and circumstances of such a subject require. With this, as with all other departments that I have made special efforts to excel in, I have found the appreciation and reward slow in coming. Whatever course my future will determine, one thing I shall do, I shall strive and aim to pursue a course that shall contribute the greatest good to the largest number possible. I know, in the ability I have acquired in the treatment of the disease that is causing the destruction of so much health, and the loss of so many teeth ultimately. I say I know, by a demonstrated practice, that I have it in my power to render a vast amount of service to my fellows; and the problem is, how to reach the people and impress them with the fact that there is a *balm* for their snfferings. How this can be *best* brought about I am not fully prepared to answer, And yet, if

I should see my way clear in the future, by casting aside all supposed professional restraints, that I may bring about a more speedy accomplishment of my purpose, I shall not shrink from it, and I give my assurance that I will strive to prove that a live professional man is one that makes his efforts concentrate toward the accumulation of such knowledge as will lift the burden of physical affliction, that hangs so like a pall over humanity; and this regardless of all the protests of a so-called code of ethics whose purposes were conceived in an antediluvian age, and have no mission in this day, I have noticed, to my sorrow, that the ones that clamor so loudly for such relics of barbarism are, by their acts, the most frequent violators of them, not so much by the use of printer's ink, as by ungentlemanly deportment, the use of unbecoming language, and their immoralities. Now, all I ask is to be judged on the basis of virtue; and may I so conduct my life that it shall write out the *unwritten* code of ethics, which shall be as pure gold refined in the crucible of human experience. For an affection for my profession I yield the palm to no one; I have, by specialized labor, daily sought its advancement in all that pertains to its upbuilding on a moral and intellectual foundation, and this I shall continue to do until I am called from my present activities into those of greater promise.

